# Novel origins of copy number variation in the dog genome

**DOI:** 10.1186/gb-2012-13-8-r73

**Published:** 2012-08-23

**Authors:** Jonas Berglund, Elisa M Nevalainen, Anna-Maja Molin, Michele Perloski, The LUPA Consortium, Catherine André, Michael C Zody, Ted Sharpe, Christophe Hitte, Kerstin Lindblad-Toh, Hannes Lohi, Matthew T Webster

**Affiliations:** 1 Science for Life Laboratory, Department of Medical Biochemistry and Microbiology, Uppsala University, Box 582, SE-751 23, Uppsala, Sweden; 2 Department of Basic Veterinary Sciences, Department of Medical Genetics, Program in Molecular Medicine, Folkhälsan Institute of Genetics, Biomedicum Helsinki, University of Helsinki, PO Box 63, 00014 Helsinki, Finland; 3 Department of Animal Breeding and Genetics, Swedish University of Agricultural Sciences, Box 597, SE-751 24 Uppsala, Sweden; 4 Broad Institute of Harvard and Massachusetts Institute of Technology, 7 Cambridge Center, Cambridge, Massachusetts 02142, USA; 5 www.eurolupa.org; 6 Institut de Génétique et Développement de Rennes, CNRS-UMR6290, Université de Rennes 1, Rennes, France

## Abstract

**Background:**

Copy number variants (CNVs) account for substantial variation between genomes and are a major source of normal and pathogenic phenotypic differences. The dog is an ideal model to investigate mutational mechanisms that generate CNVs as its genome lacks a functional ortholog of the *PRDM9 *gene implicated in recombination and CNV formation in humans. Here we comprehensively assay CNVs using high-density array comparative genomic hybridization in 50 dogs from 17 dog breeds and 3 gray wolves.

**Results:**

We use a stringent new method to identify a total of 430 high-confidence CNV loci, which range in size from 9 kb to 1.6 Mb and span 26.4 Mb, or 1.08%, of the assayed dog genome, overlapping 413 annotated genes. Of CNVs observed in each breed, 98% are also observed in multiple breeds. CNVs predicted to disrupt gene function are significantly less common than expected by chance. We identify a significant overrepresentation of peaks of GC content, previously shown to be enriched in dog recombination hotspots, in the vicinity of CNV breakpoints.

**Conclusions:**

A number of the CNVs identified by this study are candidates for generating breed-specific phenotypes. Purifying selection seems to be a major factor shaping structural variation in the dog genome, suggesting that many CNVs are deleterious. Localized peaks of GC content appear to be novel sites of CNV formation in the dog genome by non-allelic homologous recombination, potentially activated by the loss of *PRDM9*. These sequence features may have driven genome instability and chromosomal rearrangements throughout canid evolution.

## Background

The fraction of genomic variation attributable to copy number variants (CNVs) is larger than single nucleotide polymorphisms (SNPs) and yet the full extent of such structural variation is still relatively unexplored [1,2]. CNVs involve duplications, deletions or insertions of DNA segments up to several megabases in length and are responsible for significant phenotypic variation [3]. In humans, the frequency distribution of CNVs shows signals of purifying selection, suggesting that a significant proportion of CNVs have harmful phenotypic effects [1]. CNVs are associated with a number of genetic disorders, including Crohn's disease [4], psoriasis [5], osteoporosis [6], glomerulonephritis [7] and systemic lupus erythematosus [8]. However, there are also a small number of examples of CNVs that may be beneficial, such as adaptive variation in copy number of the amylase gene in response to diet [9], and variation in HIV/AIDS susceptibility [10].

A variety of mechanisms are thought to give rise to CNVs [11]. A major source of structural variation is non-allelic homologous recombination (NAHR), which occurs due to aberrant pairing of regions of extended homology. Other mechanisms involve re-joining of breaks in DNA but do not require extensive homology. In addition to this, errors in replication, such as slippage at variable number of tandem repeat (VNTR) loci or insertion of transposable elements, also generate variation in copy number. CNV formation appears to occur at higher rates in certain genomic regions termed rearrangement hotspots. In particular, CNVs associated with NAHR tend to be clustered in the genome, and CNVs are enriched in the vicinity of segmental duplications. This suggests regions of local sequence homology are hotspots of CNV formation by NAHR [12-14]. In humans, the initiation of meiotic double-stranded breaks (DSBs) is thought to begin with the binding of the protein PRDM9 to a degenerate 13-bp sequence motif [15-17]. This motif is also enriched in CNV breakpoints [2], including several involved in disease [18], which implicates DSBs formed in this way in CNV formation by NAHR.

Domestic dogs harbor an astonishing level of phenotypic variation, which is mostly apportioned into distinct breeds. The hundreds of dog breeds recognized today were formed by population bottlenecks accompanied by strong artificial selection, which has led to both their unique collections of characteristics and an increased prevalence of genetic disease. This makes the dog an ideal genetic model for uncovering the genetic basis of normal and pathogenic phenotypic variation [19]. Many traits have now been mapped in the dog genome using a variety of approaches [19-22], and structural variation is implicated in a number of these. For example, a duplication of three fibroblast growth factor (FGF) genes causes the dorsal hair ridge in Rhodesian and Thai Ridgeback dogs and predisposes to dermoid sinus [23], a duplication upstream of Hyaluronic acid synthase 2 (HAS2) is responsible for the characteristic wrinkled skin of Chinese Shar-Pei dogs and predisposes to periodic fever syndrome [24], and an insertion of an FGF4 retrogene is responsible for chondrodysplasia typical of certain breeds [25]. As in humans, much phenotypic variation is likely to be attributable to CNVs, which makes investigating them important for uncovering the genetic basis of phenotypic variation in dogs.

There is a possibility that the genomic features that promote CNV formation in dogs differ from other mammals. The dog genome differs from the majority of other mammals in that it lacks an active copy of PRDM9, which suggests that formation of meiotic DSBs is controlled differently in dogs [26]. A fine scale analysis of recombination rate variation in the dog genome indicated that, like in humans, recombination is clustered into hotspots but that, unlike in humans, these regions were strongly enriched for short regions (approximately 1 kb) of highly elevated GC content (GC peaks) [27]. This suggests GC peaks may be targets of meiotic DSBs. Interestingly, GC rich regions also seem to be involved in genome rearrangements during canid genome evolution, where they have relocated to telomeric regions [28]. This could indicate that GC peaks are important targets of NAHR and often involved in rearrangements.

Three studies have identified canine CNVs using array comparative genomic hybridization (aCGH) [29-31]. Chen *et al*. [29] used a 385,000 oligo array on nine dogs from different breed groups. They discovered 155 high confidence CNVs in 60 CNV regions. Nicholas *et al*. [30] focused on areas of segmental duplications (SDs) using single dogs from 17 breeds and a gray wolf and identified approximately 3,600 CNVs in approximately 700 overlapping regions found in two or more samples. A subsequent study [31] used aCGH with 2.1 million probes with an average density of 1 kb in nine dogs and one wolf sample and identified 403 CNVs. As expected, CNVs were found to be enriched in SDs. It was also shown that CNVs not associated with SDs were more likely to be present only once or at lower frequencies in the dataset. An additional population genetic analysis on a set of these CNVs revealed some with divergent patterns of fixation in different breeds, which could be responsible for breed-specific traits.

Despite extensive efforts to type CNVs in dogs, several questions remain about their mechanisms and effects. There is evidence that meiotic DSBs localize to different sites in dogs than other mammals [27]; does this cause a different distribution of CNVs in the genome? Is there evidence of fixed CNVs in certain breeds that may lead to breed-specific phenotypes? Can variation at CNV loci be used to delineate different breeds? Besides these questions, a comprehensive catalogue of dog CNVs would be useful to aid gene-mapping studies. Here we present the most comprehensive CNV discovery effort in dogs to date. We use a 2.1 million-probe array, as in Nicholas *et al*. [31], with probes spaced on average of 1 kb. We investigate the genome-wide extent and characteristics of CNVs in 50 dogs from 17 breeds and 3 wolves. This enables us to examine sequence features in breakpoints at high resolution and determine patterns of fixation.

## Results and discussion

### CNV discovery, genotyping and validation

We performed aCGH analysis using a 2.1M probe technology platform spanning the assayable portion of the dog reference genome with a median spacing of 1 kb. We restricted our analysis to identifying high-confidence CNVs containing at least 10 probes, which allows identification of CNVs down to approximately 9 kb in length. We assayed CNVs in 53 samples, comprising purebred dogs from 17 breeds plus 3 wolf samples. Two breeds were represented by ten unrelated individuals each to enable CNVs segregating at lower frequencies in these breeds to be identified, whereas the other breeds were represented by two individuals to maximize coverage of different breeds. A male Boxer was used as the reference sample. CNVs were analyzed in autosomes and × chromosome by comparing the ratio of signal intensities between test samples and the reference.

We identified CNVs using a three-stage procedure comprising segmentation, identification of CNV loci, and genotype calling. We first performed a comprehensive comparison of five segmentation algorithms, including NimbleGen, DNAcopy, Ultrasome, pennCNV and cghFLasso, and selected the algorithm most robust to noise (see Materials and methods and Supplementary methods in Additional file 1). This comparison identified cghFLasso as the most accurate method, while other methods resulted in excessive segmentation of the signal intensity ratio in samples with noisy data, large discrepancies in CNV numbers between samples and lack of a strong correlation between levels of variation in CNVs compared with SNPs (Supplementary methods, Tables S1 and S2 and Figures S1 to S5 in Additional file 1). We therefore used this algorithm to perform segmentation of signal intensity ratios compared to a reference for samples in our dataset.

Identification of CNV loci was performed with a method to estimate the absolute copy number compared to the reference based on fixed thresholds and chromosome-specific variance in each sample. The principle of our method is that fixed thresholds are used as a baseline cutoff, but samples with very high variance use a higher threshold whereas samples with low variance use lower thresholds (Materials and methods). We first identified segments falling above a stringent threshold, designed to exclude false positives, to identify CNV loci in single samples. The final set of CNV loci is the union of individual calls at each locus, which were merged across samples into a single CNV. The breakpoints were defined by the outermost boundaries of all individual CNV calls at each locus. After identification of loci, the genotype of each individual was inferred at each locus using less stringent criteria to determine the most likely state of each sample (described in Materials and methods ). This method allowed us to distinguish which samples exhibited deletions and duplications relative to the reference. Each change was then categorized as simple or complex based on variance between samples: changes where the mean value exceeds the threshold were considered simple, whereas changes where the mean value is less than the threshold but the mean deviation from zero exceeds the threshold were considered complex. This happens especially when the segmentation algorithm fails to discern two adjacent CNVs, of which one is a deletion and the other is a duplication (where mean value is close to zero, but mean deviation from zero is large). Loci where at least one sample exhibited a complex change from the reference were defined as complex. It is important to note that it is not possible to discern how the copies are distributed between alleles using aCGH on diploid samples, that is, distinguish if a CNV is heterozygous or homozygous.

**Figure 1:**
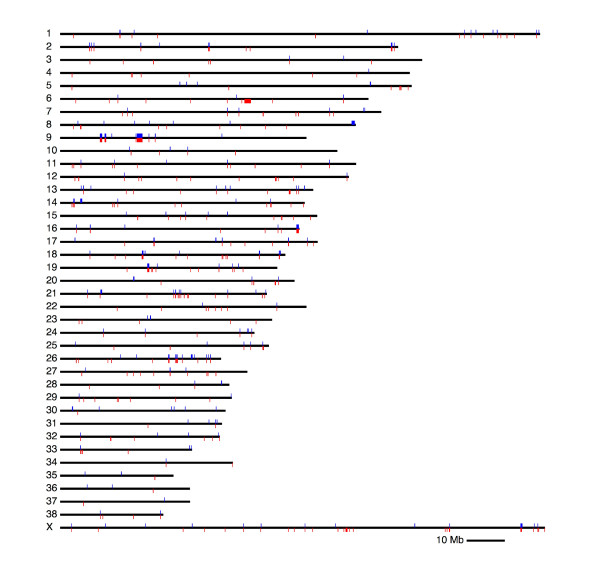
**The genomic architecture of CNVs**. Black lines represent all 38 canine autosomes and the × chromosome. Deletions are plotted as red rectangles below each chromosome and duplications are plotted as blue rectangles above each chromosome.

In total, this procedure detected 430 CNV loci distributed along the chromosomes (Figure 1). Out of these loci, 226 were classified as deletions (53%) and 104 as duplications (24%), meaning that the only variants identified at these loci were deletions or duplications, respectively, relative to the reference, whereas 100 CNV loci exhibited both deletions and duplications (23%) among samples. In addition, 77 of the loci exhibited a complex deviation from the reference in at least one sample (Table 1). Across all calls at all loci, 70.2% matched the reference, whereas 28.4% exhibited a deviation consistent with a single deletion or duplication (17.7% single deletions and 10.7% single duplications). Only 1.4% of calls were of greater magnitude (Figure S6 in Additional file 1). The finding that deletions are more numerous than duplications is generally observed in studies using aCGH [1,12,29-32]. This may reflect the greater relative difficulty of identifying duplications due to the smaller relative change in copy number (3:2 versus 2:1) and also the fact that insertions of sequence not present in the reference will not be detected. Also concordant with recent studies, duplications were found to be larger than deletions with a median size of 30 kb versus 19 kb (Table 1). This could suggest that duplications are less likely to be severely deleterious than deletions and therefore less likely to be purged by purifying selection. Alternatively, it could reflect a bias against detecting small duplications. But despite deletions being smaller, their higher incidence indicates that deletions and duplications affect similar proportions of the genome.

**Table 1:** Number of CNVs identified

	Deletions	Duplications	Both	All
Total CNV loci (complex)	226 (19)	104 (16)	100 (42)	430 (77)
Mean CNV loci per breed^a^	100.5	68.5	3.4	172.4
Mean CNV loci per sample^a^	77	53.8	0	130.9
Median size (kb)	19	29.5	27.8	24.3

Deletions are defined as CNV loci that only exhibit reduction in copy number relative to the reference assembly. Duplications are loci that only exhibit increased copy number. 'Both' refers to loci that exhibit both deletions and duplications. ^a^Based on 15 breeds with 2 samples.

On average, 130.9 loci (30%) differ from the reference per sample, and among breeds with two samples an average of 172.4 loci (40%) differ from the reference in one or both of the samples (Table 1). In total 50 (12%) CNVs were detected only once in the dataset. This is an average of one singleton per sample, and is consistent across samples from breeds with ten and two samples. We observe one CNV that differs from the reference in all samples, indicating that it is a singleton in the reference (chromosome 15, 57.86 to 57.88 Mb). This corresponds well with the number of singletons found in other samples, suggesting these numbers are accurate. However, this contrasts with a recent study by Nicholas *et al*. [31] using the same array, which identified 403 CNV loci in nine dogs and a wolf, of which 260 (65%) were detected in a single sample (an average of 26 per sample). This previous study used only one sample per breed, which makes identifying CNV loci as singletons more likely. However, the fact that we also find that most CNV loci are shared between breeds suggests that differences in CNV calling contribute to differences in CNVs found in each study.

We validated our set of CNVs using two complementary methods: quantitative PCR (qPCR) and analysis on the CanineHD high-density SNP genotyping array. Because of the high overlap (50%) with previously identified and confirmed CNVs (see 'Distribution and genomic effects of CNVs'), we focused our validation on novel CNVs detected only in our study, and performed qPCR on four loci ranging in size from 17 to 80 kb. A total of 53 sample-locus combinations were tested and 3 tests did not match the state predicted from aCGH (1 false positive and 2 false negatives; 94% concordance).

All of the samples used for aCGH were also genotyped on the Illumina CanineHD array, which contains >174,000 probes designed for assaying SNPs with an average spacing of 13 kb [33]. The probe density of this more than ten times sparser array permits identification of larger CNVs (greater than approximately 100 kb with our filters) compared to the aCGH chip. In total, 13 CNV loci passed our filtering procedure and had enough probes on the SNP array to be used in the validation procedure (Table S3 in Additional file 1). These 13 CNVs, with an average size of 266 kb, included 8 singletons, 3 two-sample CNVs and 2 CNVs at midrange frequency. In total there were 6 mismatches between calling on the aCGH and CanineHD SNP array among 689 genotypes (1 false positive and 5 false negatives), which is a correspondence of >99% between individual genotypes. This suggests that the genotyping error is smaller than the 6% estimated from the qPCR, at least for large CNVs.

### Distribution and genomic effects of CNVs

**Figure 2:**
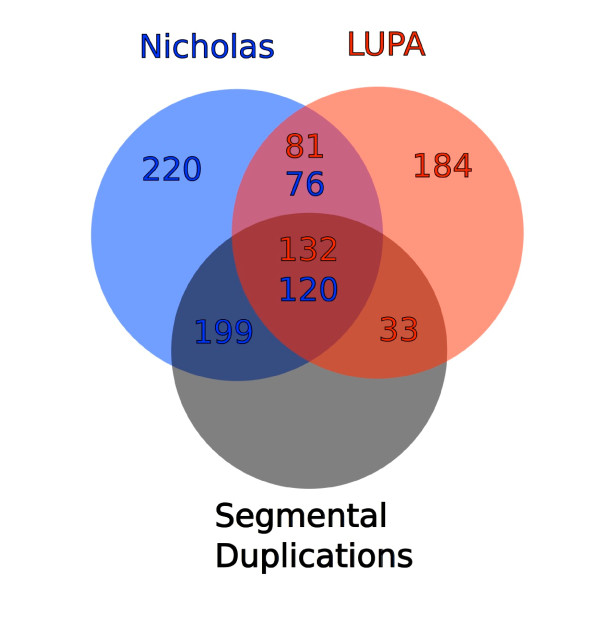
**Comparison of LUPA dataset with a summary of CNVs presented by Nicholas *et al***. [31]**and a list of segmental duplications in dog **[53]. The Venn diagram shows the number of overlapping CNVs from the datasets. Since the datasets contain different entries, numbers are colored according to which dataset the counted entries belong to. For example, 196 of Nicholas et al.'s CNVs overlap with 213 of our (LUPA) CNVs, and 120 and 132, respectively, of these also overlap with segmental duplications.

We compared our dataset to CNVs previously identified in dog in multiple studies merged and augmented by Nicholas *et al*. [31]. We identify 216 overlaps, which contain 196 (of 615) of previously identified CNVs and 213 (of 430) of ours (Figure 2). This overlap is highly significant compared to random redistributions of CNVs in the genome (*P *< 0.001). The reason that some CNVs do not overlap between studies is likely to be a combination of differences in breed selection, sample size, array resolution, genotyping algorithms and errors. Furthermore, we generated a canine segmental duplication map using a modified version of the method of Bailey *et al*. [34] (described in [35]), to which both datasets were mapped. The Venn diagram in Figure 2 shows that more than a third (165) of our CNVs overlap these SDs and more than half (319) of Nicholas' CNVs overlap SDs, which is a highly significant overlap (*P *< 0.001, random redistribution test) considering <5% of the dog genome is composed of recent SDs. The high proportion of CNVs in SDs together with our non-targeted approach indicates a large involvement of SDs in CNV formation as indicated by previous studies. However, it should also be noted that a small proportion of SDs are likely not fixed in the dog genome and may actually be CNVs.

The largest CNV locus we identified in the dog genome is located at 48.5 to 50.0 Mb on chromosome 6. The pattern of variation in this region is consistent with a deletion in all samples except both Labradors and one Boxer, which match the reference sequence. Inspection of probe intensities in this region on the CanineHD array shows a pattern consistent with the presence of duplicated sequence in these Labrador and Boxer samples. Considering the high frequency of the deletion allele, it is most likely that the duplication allele is the derived state. The CNV encompasses a gene-sparse region downstream of the RNA-binding region gene *RNPC3 *and upstream of the collagen genes *COL11A1 *and *COL5A1*.

The second largest locus is a complex CNV on chromosome 9 between 20.1 and 21.6 Mb. There are no RefSeq annotated dog genes in this region, although it is orthologous to the human genes *VPS13D*, *RDM1*, *ARHGAP27 *and members of the *LRRC37 *family. These genes are not co-located in human, so this region must represent synteny to multiple loci. *RDM1 *is near members of the *TBC1D3 *family, which shows primate-specific expansion via SDs on chromosome 17, and is present in a majority of human-specific breakpoints of conserved synteny to mouse [36]. Both *LRRC37 *and *ARHGAP27 *are orthologous to regions flanking an inversion in the human genome at 17q21.31, thought to have arisen through NAHR between large blocks of flanking SDs, which are distributed throughout chromosome 17 and contain the *LRRC37 *gene family [37]. This region is associated with a micro-deletion leading to mental retardation and has undergone multiple complex rearrangements during primate evolution. The finding that this region is also a large and complex CNV in dogs suggests that it may be a region of instability across a wide range of mammalian evolution. Both of the two discussed loci were also present in previous dog aCGH reports [29-31].

CNVs overlapping SDs occur at higher frequencies in the population (*P *< 0.001), are more likely to be complex (*P *< 0.001), tend to be longer (*P *< 0.001), and are more likely to overlap genes (*P *< 0.001) than CNVs not associated with SDs (bootstrapping used for all significance tests), which confirms previous observations [31]. Both singletons (80%) and breed-specific CNVs (85%) are more likely to fall in the category of CNVs not overlapping SDs. Non-SD CNVs have an average frequency of 0.24 (0.33 of breeds), whereas CNVs inside of SDs have an average frequency of 0.4 (0.56 of breeds), which is almost twice as high (*P *< 0.001). Complex CNVs are preferentially observed in SDs, where every third CNV is complex, compared to less than 10% complex CNVs outside SDs. These observations may reflect a higher mutation rate of CNVs in SDs, with recurrent events around the same genomic location leading to both higher frequencies and more complexity, but could also reflect the involvement of SDs in more complex rearrangements. CNVs in SDs are also larger than non-SD CNVs; with a median size of 40 versus 20 kb. The increased frequency and complexity of CNVs in SDs may reflect the dynamic nature of SDs, and that these CNVs have arisen from overlapping but distinct events.

We next examined the functional effects of CNVs by identifying genes they overlap. In order to use a high-confidence gene set, 24,232 dog genes annotated under Ensembl ID were filtered with the g:Orth tool from the g:Profiler website to extract only human-dog 1:1 orthologs. Out of 15,258 1:1 orthologous genes, 130 genes (0.85%) are overlapped either completely or partially by 90 CNVs (out of 430 total CNVs). This number is significantly less than predicted by chance (*P *< 0.001, random redistributions per chromosome). A great proportion of the affected genes, 53 (41%), had their entire coding sequence covered by a CNV (Table 2). This could suggest that CNVs overlapping genes are more likely to have deleterious effects. A test of the size distribution of CNVs affecting genes revealed that they are larger, with median size of 36 versus 24 kb (bootstrapping, *P *< 0.005). They also show a slight tendency towards lower frequencies, although the difference is not significant (bootstrapping, *P *< 0.1).

**Table 2:** Number of CNV loci covering genomic regions

			Intragenic			
			
					Partial gene	
					
	Total	Intergenic	Total	Whole gene	Total	Stop codon
Total	430	340	90	31	67	29
Deletion	226	198	28	8	21	4
Duplication	104	62	42	13	33	19
Both	100	80	20	10	12	6
Deletion:duplication ratio	2.17	3.08	0.67	0.62	0.64	0.21

All groups are non-overlapping. Subcategories are not additive as a CNV can span several genes and harbor both deletions and duplications in different samples.

In addition to their paucity, CNVs overlapping genes are characterized by a much higher proportion of duplications than deletions (Table 2). In intergenic CNVs, deletions are more than three times as common as duplications, whereas within genes they occur at a proportion of two-thirds compared with duplications (*P *= 3.1 × 10^-8^; Fisher's exact test). This pattern is even more pronounced in intragenic CNVs that are predicted to remove a stop codon, where deletions occur at a proportion of 0.2 relative to duplications, which is significantly lower than other intragenic CNVs (*P *= 0.009; Fisher's exact test). This tendency for duplication enrichment among stop codons has previously been detected in humans [1], and suggests a strong deleterious effect of removal of stop codons.

The set of 130 1:1 orthologous genes overlapping CNVs were scanned for enrichment of gene ontology (GO) categories against a background of all 15,258 1:1 human-dog orthologs (Table 3). This gene set was chosen because of the higher accuracy of annotations for human genes. For this purpose the g:GOSt tool from g:Profiler website was used. The most significantly enriched term in each domain was the biological process 'homophilic cell adhesion' (*P *= 7.31 × 10^-13^), the cellular component 'integral to membrane' (*P *= 6.07 × 10^-5^) and the molecular function 'olfactory receptor activity' (*P *= 6.22 × 10^-6^). Cell adhesion also appears as a strongly enriched category among human CNVs [1,32]. In our dataset, CNVs are also enriched for genes involved in the Kyoto Encyclopedia of Genes and Genomes (KEGG) pathway 'olfactory transduction' (*P *= 5.04 × 10^-3^). Analyzing simple deletion and simple duplication loci separately reveals a difference in the kind of genes they overlap: no functional categories are inferred to be enriched in both deletions and duplications. For example, the 'homophilic cell adhesion' category is only enriched in deletions, whereas 'olfactory receptor activity' is only enriched in duplications. The enrichment of specific GO categories could reflect changes in patterns of selective constraint in dogs [38], positive selection for dog-specific traits, or tolerance of certain gene categories to deletions or duplications.

**Table 3:** Enriched gene ontology categories in genic CNVs

*P*-value^a^					Number of genes			
				
All CNVs	Deletions	Duplications	GO ID	Domain	All	Deletions	Duplications	Gene ontology term
9.48e-02	-	**3.12e-03**	GO:0032787	BP	5	0	5	1. Monocarboxylic acid metabolic process
**5.00e-06**	**6.32e-04**	1	GO:0022610	BP	14	7	1	1. Biological adhesion
**5.00e-06**	**6.32e-04**	1	GO:0007155	BP	14	7	1	2. Cell adhesion
**2.67e-11**	**1.36e-06**	1	GO:0016337	BP	14	7	1	3. Cell-cell adhesion
**7.31e-13**	**2.37e-07**	1	GO:0007156	BP	14	7	1	4. Homophilic cell adhesion
**3.56e-03**	6.29e-02	1	GO:0071944	CC	18	8	2	1. Cell periphery
**2.68e-04**	1.76e-01	1	GO:0016020	CC	47	16	20	1. Membrane
**2.15e-03**	**4.88e-02**	1	GO:0005886	CC	18	8	2	2. Plasma membrane
**3.37e-04**	6.08e-01	3.64e-01	GO:0044425	CC	40	13	19	1. Membrane part
**8.74e-05**	2.49e-01	3.45e-01	GO:0031224	CC	39	13	18	2. Intrinsic to membrane
**6.07e-05**	2.17e-01	2.92e-01	GO:0016021	CC	39	13	18	3. Integral to membrane
5.97e-02	1	**3.82e-02**	GO:0004872	MF	16	4	12	1. Receptor activity
-	1	**2.66e-02**	GO:0038023	MF	18	3	11	2. Signaling receptor activity
**1.96e-02**	1	**1.27e-02**	GO:0004888	MF	16	3	11	3. Transmembrane signaling receptor activity
**1.49e-03**	1	**5.21e-04**	GO:0004930	MF	15	3	11	4. G-protein coupled receptor activity
**6.22e-06**	1	**9.88e-06**	GO:0004984	MF	12	2	9	4. Olfactory receptor activity
**3.18e-04**	**7.11e-05**	1	GO:0005509	MF	17	10	1	1. Calcium ion binding
5.20e-01	-	**9.98e-03**	GO:0005506	MF	6	0	6	1. Iron ion binding
**5.04e-03**	1	**6.44e-03**	KEGG:04740	ke	7	1	5	1. Olfactory transduction

^a^Significant *P*-values are in bold. *P*-values were determined using a hierarchical multiple testing procedure. Indented numbers before the GO terms indicate relative depth of the term in the hierarchy. BP, biological process; CC, cellular component; MF, molecular function; ke, KEGG pathway.

### Mechanisms of CNV formation

We searched for repeats that were enriched close to breakpoints of CNVs, calculating the observed to expected ratio to identify over-represented motifs. There is uncertainty in precisely defining breakpoint location because of smoothing of probe intensities and experimental noise. Each breakpoint location was defined as a 10 kb window to account for this imprecision. The list of known repeats was downloaded from the RepeatMasker track of the UCSC genome browser. Nearly all CNV breakpoints overlap some repeat family, with L1, ERV[1/L], RNA and satellite DNA being overrepresented (Table S4 in Additional file 1). The last two types involve fewer than ten breakpoints, and cannot be considered a significant contribution to CNV formation. However, >96% of the CNV breakpoints contain LINE elements, an excess of 36% compared with expected coverage, and almost 65% of the CNV breakpoints contain a long terminal repeat (includes the endogenous retrovirus (ERV) family) entry. We found a 1.5-fold excess of L1 elements in CNV breakpoints. Interestingly this excess seems to be most prominent for younger L1 repeats (Table 4). This follows the pattern of CNVs and SDs detected in humans [32], where the likelihood of a SD being associated with a CNV was highly correlated to its sequence similarity to the duplicated copy, as younger L1 elements are likely to have increased levels of sequence homology with each other.

**Table 4:** Excess of L1 repeats in CNV breakpoints

Name	Excess^a^	Divergence^b^	Length (bp)	Number of repeats in genome	Number of repeats in breakpoints	Number of breakpoints with repeats
L1_Cf	3.92	0.035	1,192	16,127	178	137
L1_Canis	2.54	0.088	799	72,587	678	309
L1_Canid	1.87	0.144	534	42,070	254	135
L1_Carn	1.25	0.168	525	132,806	652	275
Eutheria	0.91	0.246	359	589,155	2046	590

^a^The proportion of repeat coverage in breakpoints versus whole genome. ^b^Average divergence of the repeat from its reference in RepBase.

The pattern of L1 enrichment together with the significant overlap between SDs and CNVs is consistent with a large contribution of NAHR, which is known to operate on highly similar copies, and supports the generation of CNVs in duplicated regions and regions with mobile element insertions. L1 transduction, where additional 3' flanking sequence is transferred to a new genomic location together with an L1 insertion, may also contribute to this enrichment. We do not find any enrichment of CNV breakpoints around SINEs. This is in concordance with some studies in humans [39,40] that find associations with L1 but not *Alu *at CNV breakpoints. This may suggest that LINE elements promote structural variation through NAHR more strongly than SINEs in the dog genome.

**Figure 3:**
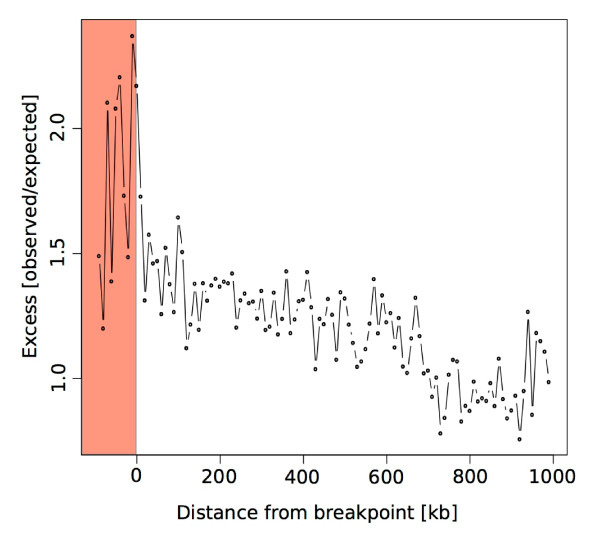
**Enrichment of GC peaks around CNV breakpoints**. The strongest excess of GC peaks compared to random expectations is observed close to CNV breakpoints and decays with increasing distance from the breakpoint. The midpoint of the breakpoint is at position 0, the shaded area to the left of this represents points within the CNV, and the area to the right represents flanking sequence.

As GC peaks are enriched in recombination hotspots in the dog genome and may be important for formation of DSBs [27], we searched for enrichment of this sequence feature within CNV breakpoints. We first defined GC peaks as in Axelsson *et al*. [27], where a GC peak is recorded when a 500-bp sliding window centered in a 10 kb background sliding window has a 1.5-fold increase in GC content. We then performed randomization tests to analyze enrichment of GC content in breakpoints. We found a more than two-fold enrichment in CNV breakpoints (*P *< 0.001), which rapidly decays with increasing distance from the breakpoint, both within and outside of the CNV (Figure 3). We also found an enrichment of CpG islands and gaps in CNV breakpoints (*P *< 0.001; Table S5 in Additional file 1). CpG islands are usually GC-rich, and the great overlap with GC peaks is somewhat expected. Gaps in the reference genome assembly have shown an extremely high likelihood of being associated with CNVs in human [32]. In dog, gaps tend to correlate with high GC content because these regions are less well captured during the genome sequencing, and many of them could qualify as GC peaks if they were fully characterized. The association between CNV breakpoints and GC peaks in dogs suggests that CNV breakpoints may often occur at recombination hotspots, where DSBs have a tendency to form, followed by their repair by NAHR.

The association with GC peaks and CNVs has not been reported in other species. In humans, a 13-bp sequence motif targeted by the PRDM9 protein is strongly associated with recombination hotspots, which also associates with CNVs [18]. In dogs, the *PRDM9 *gene is inactive and recombination hotspots are strongly associated with GC peaks. The association between GC peaks and CNV breakpoints may, therefore, be an additional consequence of the death of *PRDM9 *[27]. There is a strong overlap between CpG islands and GC peaks, and both are enriched close to CNV breakpoints. This could indicate that non-methylated DNA promotes DSB formation that leads to structural variation. However, it is also possible that it is simply GC-richness that promotes recombination, or that the peaks of high GC content are mainly a consequence of elevated recombination rate due to GC-biased gene conversion rather than its cause, which are detected as CpG islands regardless of methylation [41]. These results are particularly interesting in light of the suggestion that GC-rich regions have acted as novel target sites of chromosomal fissions during canid evolution [28].

We also searched for regions of extended perfect homology between pairs of breakpoints flanking CNV loci. Of the 430 CNV loci, 86 have runs of perfect sequence homology greater than 75 bp and 11 have stretches longer than 1 kb. The mean length of runs of perfect homology between breakpoints is 126.8 bp. We tested the significance of these stretches of homology by simulating 1,000 datasets with the same number of loci and distance between breakpoints, located at randomly chosen positions on the same chromosomes. The mean length of homology in the simulated dataset was 35.0 bp, with none of the simulated means exceeding the observed one (*P *< 0.001). These findings provide additional support for an important role of NAHR in dog CNV formation. Surprisingly, the mean length of homology between breakpoints of CNVs overlapping SDs is slightly shorter than those outside of SDs (133.1 bp compared with 116.6 bp). This suggests that NAHR is not restricted to segmental duplications and may be equally or more common outside of them.

### CNV distribution among breeds and samples

We next analyzed the distribution of CNVs among breeds (Table 5). The majority (341, 79%) of CNVs were found in several breeds, while 89 CNVs (21%) were breed-specific. The breed-specific CNVs are larger (32 kb) than CNVs found in multiple breeds (22 kb) (*P *< 0.005, bootstrapping). Some variation in the number of CNVs between samples was seen both within and between breeds. Notably, the total number of CNVs identified in Boxers was lower than in any other breed, with an average of 64.5 loci different from the reference per sample, largely due to the reference being a Boxer. Of the remaining breeds, the average number of CNVs per sample varied from 116.5 (Swedish Elkhound) to 160 (English Springer Spaniel). On average, a sample differs from the reference at 130.9 CNV loci, of which 2.8 are specific to that breed. Fewer than 6% of CNVs found in any one breed are specific to that breed. These patterns are broadly consistent in the wolf samples, which exhibit a slightly lower than average number of CNVs per sample. On average, 2 dogs from the same breed differ at 83.1 CNV loci whereas 2 dogs from different breeds differ at 103 CNV loci.

**Table 5:** CNVs identified in each breed and sample compared to the reference genome

		Total CNV loci per breed			Average CNV loci per sample		
			
Breed	Samples	Total	MB	BS	Total	MB	BS
Border Terrier (BTe)	2	156	154	2	128.5	126.5	2
Boxer (Box)	2	103	102	1	64.5	64	0.5
Cavalier King Charles Spaniel (CCS)	2	189	182	7	148.5	143.5	5
Chihuahua (Chi)	2	181	178	3	142	140.5	1.5
Dachshund (Dac)	2	180	172	8	131	127	4
English Cocker Spaniel (ECS)	2	166	165	1	129	128.5	0.5
English Springer Spaniel (ESS)	2	214	207	7	160	156	4
Finnish Spitz (FSp)	2	186	176	10	148.5	140.5	8
German Shepherd (GSh)	2	163	160	3	126	123.5	2.5
Labrador Retriever (LRe)	2	170	168	2	129	128	1
Nova Scotia Duck Tolling Retriever (NSD)	2	193	190	3	147.5	145.5	2
Poodle (Pdl)	2	182	180	2	130	129	1
Sarloos (Sar)	2	175	168	7	135	130	5
Schnauzer (Sch)	2	169	163	6	127	123.5	3.5
Swedish Elkhound (Elk)	2	159	158	1	116.5	115.5	1
**Average (2-sample breeds)**	**2**	**172.4**	**168.2**	**4.2**	**130.9**	**128.1**	**2.8**
Golden Retriever (GRe)	10	264	255	9	121.6	118.8	2.8
Irish Wolfhound (IrW)	10	229	215	14	136.2	127.7	8.5
**Average (10-sample breeds)**	**10**	**246.5**	**235**	**11.5**	**128.9**	**123.3**	**5.7**
Wolf (Wlf)	3	163	160	3	96.3	95.3	1

MB, multi-breed (CNVs found in more than one breed); BS, breed-specific (CNVs found in only one breed).

A more detailed picture of polymorphism in breeds with two samples is given in Table 6. We find that an average of 172.4 CNV loci (40%) are observed in one or both of the samples in any one breed and an average of 4.2 (2.4%) are only found in that breed (private CNVs). Similar numbers of CNVs are found in one sample (polymorphic) or both samples (fixed) of these breeds. An average of one private CNV is fixed (found in both samples) in the breeds, although with a small sample size, the majority of these are likely to be polymorphic.

**Table 6:** Polymorphic CNVs in breeds with two samples

		Loci matching	Shared loci between breeds			Private loci single breeds		
				
Breed	Samples	reference	Total	Polymorphic	Fixed	Total	Polymorphic	Fixed
Border Terrier	2	274	154	55	99	2	0	2
Boxer	2	327	102	76	26	1	1	0
Cavalier King Charles Spaniel	2	241	182	77	105	7	4	3
Chihuahua	2	249	178	75	103	3	3	0
Dachshund	2	250	172	90	82	8	8	0
English Cocker Spaniel	2	264	165	73	92	1	1	0
English Springer Spaniel	2	216	207	102	105	7	6	1
Finnish Spitz	2	244	176	71	105	10	4	6
German Shepherd	2	267	160	73	87	3	1	2
Labrador Retriever	2	260	168	80	88	2	2	0
Nova Scotia Duck Tolling Retriever	2	237	190	89	101	3	2	1
Poodle	2	248	180	102	78	2	2	0
Sarloos	2	255	168	76	92	7	4	3
Schnauzer	2	261	163	79	84	6	5	1
Swedish Elkhound	2	271	158	85	73	1	0	1
**Average**	**2**	**257.6**	**168.2**	**80.2**	**88**	**4.2**	**2.9**	**1.3**
Wolf	3	267	160	121	39	3	3	0

Total, number of CNVs observed in the breed; Polymorphic, average number of polymorphic CNV sites between samples of the breed; Fixed, number of CNVs found in both samples of the breed.

**Figure 4:**
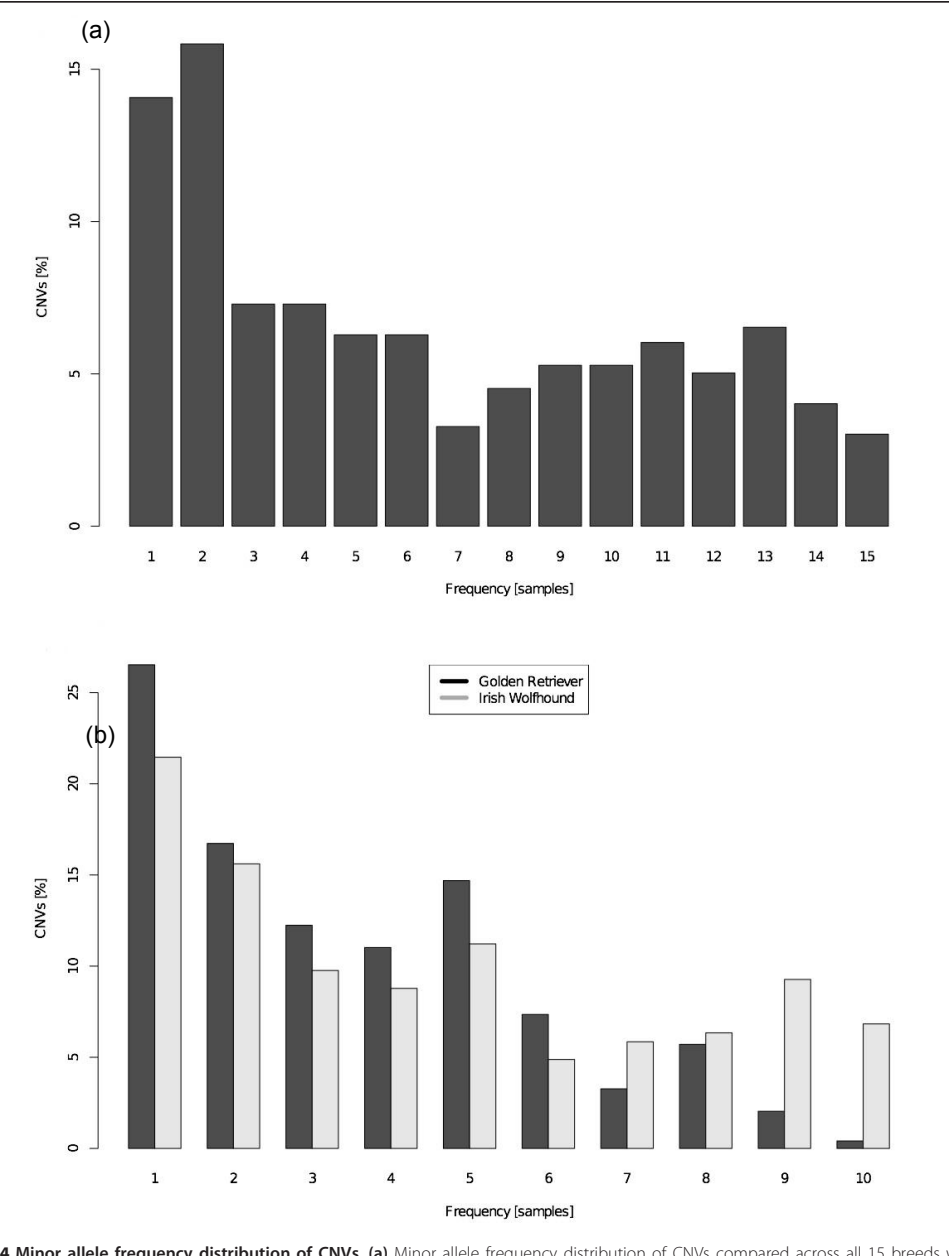
**Minor allele frequency distribution of CNVs**. **(a) **Minor allele frequency distribution of CNVs compared across all 15 breeds with a sample size of two. **(b) **Minor allele frequency distribution of CNVs within the two breeds with a sample size of ten.

To assess the frequency distribution of CNVs among samples, we used their presence in all samples to build a site frequency spectrum. Figure 4a shows the minor allele frequency distribution across all samples in two-sample breeds. There is a marked drop in frequency above two samples, which can be attributed to the higher within compared to between breed fixations, where two samples are present per breed, making it less likely that three or more samples share a CNV. A broadly similar frequency distribution is seen within breeds. Figure 4b shows the allele frequency distribution with ten genotyped individuals analyzed on a breed basis (identity of the minor allele is defined from the two-sample breeds of the entire dataset).

The two breeds for which ten individuals were analyzed were selected for their large (Golden Retriever) and small (Irish Wolfhound) population sizes, respectively. We first attempted to see how many CNVs were present in all ten dogs of a breed, suggesting fixation (Table 7). For Golden Retrievers, 20 CNVs were present in all dogs, and for Irish Wolfhounds, 38 CNVs appeared fixed, possibly reflecting the slightly higher degree of inbreeding in Irish Wolfhounds. A much smaller number of breed-specific loci were identified, and no cases of breed-specific fixed CNVs were identified in these deeply sampled breeds. The relative lack of breed-specific fixed CNVs suggests that instances of those involved in breed-specific phenotypes must be rare. Overall, the CNV frequency distributions appear qualitatively similar to those expected for neutral polymorphisms.

**Table 7:** Fixed CNVs in breeds with ten samples

	Loci matching	Shared loci between breeds			Private loci in single breed		
			
Breed	reference	Total	Polymorphic	Fixed	Total	Polymorphic	Fixed
Golden Retriever	166	255	235	20	9	9	0
Irish Wolfhound	203	215	177	38	14	14	0
**Average**	**184.5**	**235**	**206**	**29**	**11.5**	**11.5**	**0**

Total, number of CNVs observed in the breed; Polymorphic, average number of polymorphic CNV sites between samples of the breed; Fixed, number of CNVs found in both samples of the breed.

We scanned our dataset for CNVs that were fixed for one allele in some two-sample breeds and another allele in all other two-sample breeds (that is, are not polymorphic in any breed). There are 24 such CNVs, of which all but one is specific to a single breed. This CNV is a 12.7 kb deletion (located at 55.5 Mb on chromosome 6) shared by Cavalier King Charles Spaniel and English Springer Spaniel, which overlaps the gene *DPYD*, which encodes the enzyme dihydropyrimidine dehydrogenase (DPD) involved in pyrimidine catabolism. Examples of breed-specific fixations include a 32.7 kb deletion on chromosome 28 in German Shepherds downstream of *DUSP10*, which is involved in immune responses and mediates various physiological processes, and a 18.1 kb deletion on chromosome 21 in Finnish Spitz immediately downstream of *CYP2R1 *(cytochrome p450, family 2, subfamily R, polypeptide 1) and overlapping *PDE3B *(phosphodiesterase 3B, cGMP-inhibited). These regions are good candidates for governing breed-specific characteristics, although further investigation is necessary to determine if they are really fixed, or have any phenotypic effect.

### Breed relationships

**Figure 5:**
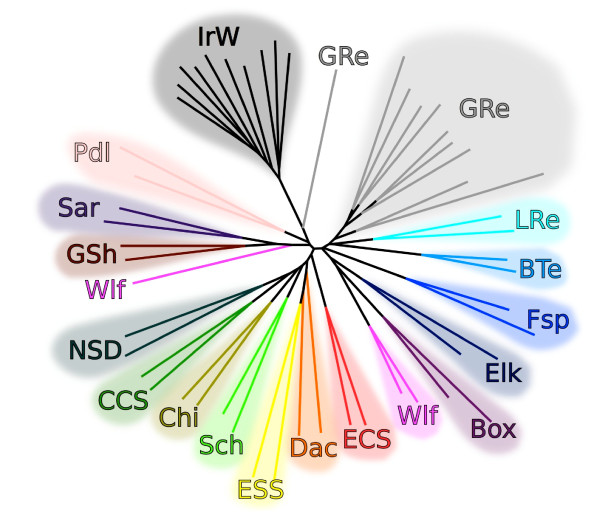
**Neighbor-joining tree based on allele sharing at CNV loci**. Box, Boxer; BTe, Border Terrier; CCS, Cavalier King Charles Spaniel; Chi, Chihuahua; Dac, Dachshund; ECS, English Cocker Spaniel; Elk, Swedish Elkhound; ESS, English Springer Spaniel; Fsp, Finnish Spitz; GRe, Golden Retriever; GSh, German Shepherd; IrW, Irish Wolfhound; LRe, Labrador Retriever; NSD, Nova Scotia Duck Tolling Retriever; Pdl, Poodle; Sar, Sarloos; Sch, Schnauzer; Wlf, Wolf.

We explored the extent to which patterns of CNV variation can be used to infer population structure between breeds. Based on the proportion of shared CNVs between each pair of samples, a neighbor-joining phylogeny was constructed (Figure 5). With a few exceptions, all samples clustered together with samples from the same breed. This indicates that the CNVs have a strong phylogenetic signal, grouping dogs into breeds as shown by large-scale SNP analyses [33,42]. Ability to construct this tree demonstrates the high accuracy of dataset and high congruency with SNP data. Notably, one of the wolf samples clusters on a branch leading to Sarloos (a wolf hybrid) and German Shepherd, as observed in a previous SNP analysis [33]. There is also some evidence for clustering by breed type as demonstrated by vonHoldt *et al*. [42]. One cluster contains spaniels (English Cocker Spaniel, English Springer Spaniel, Cavalier King Charles Spaniel), scent hounds (Dachshund) and toy dogs (Chihuahua), and another contains retrievers and terriers (Golden Retriever, Labrador Retriever, Border Terrier). However, there are exceptions present, and in general, this analysis is limited by a smaller number of loci and less precise calling than the studies based on SNP arrays.

## Conclusions

This study provides insights into the mutational mechanisms and functional effects of CNVs in the dog genome. Our results suggest that many CNVs are generated by NAHR events directed towards peaks of GC content, which is consistent with observations that these sequence features are also enriched in dog, but not human, recombination hotspots. Hence, GC peaks may represent novel sites of elevated recombination and genome instability in dogs. This shift in recombinational activity towards GC peaks in dogs is likely to be due to the lack of a functional copy of the *PRDM9 *gene, which initiates recombination at separate specific sequence motifs in humans. In support of a strong role of NAHR in dog CNV formation, we also identify associations between CNV breakpoints and L1 elements and long stretches of sequence homology. We also show that dog CNVs are affected by the signal of purifying selection and identify candidate CNVs for involvement in breed-specific characteristics. This comprehensive catalogue of CNVs will be useful for future studies to uncover the genetic basis of complex traits in dogs.

## Materials and methods

### Sample collection

EDTA-blood was collected as part of the LUPA project [43] from pedigree dogs around Europe and USA with owners' consents: a total of 50 dogs from 17 breeds including 2 unrelated individuals from the breeds Border Terrier, Boxer, Cavalier King Charles Spaniel, Chihuahua, Dachshund, English Springer Spaniel, English Cocker Spaniel, Finnish Spitz, German Shepherd, Labrador Retriever, Nova Scotia Duck Tolling Retriever, Poodle, Sarloos Wolfhound, Schnauzer and Swedish Elkhound; and 10 unrelated individuals from Golden Retriever and Irish Wolfhound breeds. In addition, three gray wolves from Belarus, Spain and Finland, respectively, were included. A Finnish male Boxer was used as the reference sample. Genomic DNA was purified using commercial purification kits and the quality of the DNA was analyzed by spectrophotometry and agarose gel electrophoresis prior to the hybridization experiment.

### Discovery and genotyping

aCGH was used to detect DNA copy number alterations using NimbleGen's canFam2 Whole Genome CGH oligo array platform with 2.1 million probes on a single slide and a median probe spacing of 1 kb. The array design is based on the annotated CanFam2.0 genome sequence of a female Boxer genome. The isothermal 50-75mer probes were evenly distributed throughout the unique sequence of the genome. The genomic DNA samples were sent to NimbleGen's service facility where the hybridizations were performed in a two-color format according to Selzer *et al*. [44]. Copy number was quantified from the fluorescence ratios of the two dyes. NimbleGen conducted the initial data processing from normalization to signal calling and quantification. Ratios were log2 transformed, and positive log2 ratios indicate gains and negative log2 ratios indicate loss of copy number. Copy number for each called CNV was calculated as 2^(1+mean log2ratio) ^and rounded to whole numbers.

CNV calling was conducted in three stages: smoothing, segmentation and selection. Prior to segmentation and selection, triangular smoothing was done on the ratios, which implements an 11-point weighted smoothing function along the chromosomes. This is equivalent to several passes of fewer-point rectangular smoothings (unweighted sliding-average), and is more effective at reducing high-frequency noise in the signal. Segmentation was done with the R package cghFLasso to segment a continuous distribution of intensity ratios into discrete regions of consecutively different ratios. From these putative CNV segments, those with significant deviation are selected as the final set of CNVs. The selection was performed in three stages: first, ascertain CNV locations per sample using a stringent threshold (discovery threshold); second, consolidate the calls from all individuals by mapping them onto one 'master genome' to get merged general CNV locations; and third, decide the state of the general CNVs in each breed using a less stringent threshold (genotyping threshold) to assign individuals to diploid copy number classes. Noisy data were handled by recalculating the ratios into values of standard deviation from a theoretical normal distribution of ratios. These were used to allow samples with little noise to utilize lower thresholds. The two thresholds were then individually chosen on chromosome basis from a fixed log2 ratio and a standard deviation value.

The fixed genotyping ratio was set to correspond to a deviation of 0.5 copies from the reference, while the fixed discovery ratio was set to the slightly higher 0.65 copies deviation. The standard deviation genotyping value was chosen to include 5% of the data points, while the standard deviation discovery value was chosen to include 0.1% of the data points, from the theoretical normal distribution (any copy number variants should be clearly distinct from the distribution). These were chosen so that a similar number of samples used the fixed values and the standard deviation values. The discovery threshold was picked as the maximum of these two numbers, while the genotyping threshold was picked as the minimum of the two numbers. This means that no CNVs are identified in the first stage if the deviation from the reference is below 0.65 copies, and in the final stage all CNVs with a deviation above 0.5 copies from the reference are identified as CNVs.

### Validation

aCGH results were experimentally validated by qPCR in randomly selected CNV loci. Relative copy numbers of the selected regions were determined in comparison to the reference sample (Finnish male Boxer). Regions were selected based on the aCGH profiles across breeds. qPCR experiments were performed on ABI Prism 7500 Fast instrument (Applied Biosystems, Stockholm, Sweden) using SYBR Green detection chemistry according to the manufacturer's instructions. Primers (available upon request) were designed inside CNVs using Primer3 and NCBI primer design programs. Each assay was performed in triplicate using 20 μl reactions containing 10 μl of qPCR master mix (Roche, Stockholm, Sweden), 10 nM concentration of dNTPs, 250 nM concentration of forward and reverse primer and 10 ng of genomic DNA. Amplification was performed under the following conditions: one cycle at 50°C for 2 minutes, one cycle at 95°C for 10 minutes, 40 cycles at 95°C for 15 seconds and 62°C for 45 seconds. Beta-actin and GAP175 were used as controls. Serial dilutions were performed for each assay to estimate the PCR efficiency (E) prior to analysis. The ddC_T _method was used for the quantification of copy numbers in test individuals relative to the same reference Boxer sample used in the aCGH experiments. The C_T _values for each set of triplicates were averaged and adjusted for PCR efficiency (E) as log2(E^CT^). The C_T _values were then normalized against the control primers. The relative copy number for each site was calculated as 2^-(t-r)^, where t = normalized C_T _for the test sample and r = normalized C_T _for the reference sample.

Large CNVs, by virtue of their potential functional impact, were validated in an additional validation step. The same set of individuals was genotyped according to manufacturer's instructions on the CanineHD 170K SNP array (Illumina) with a resolution of 1 SNP per 13 kb. The GenomeStudio V2010.3 software package (Illumina) was used to obtain normalized total signal intensity, Log R ratio, and B allele frequencies for all SNPs according to the manual and Peiffer *et al*. [45]. Exported Log R ratio and B allele frequencies for every SNP were used in the subsequent CNV calling. The CNV calling was performed using QuantiSNP [46]. Default settings in QuantiSNP were used, that is, L = 2M, expectation-maximization iterations =10, and the parameter file levels-hd.dat. Samples having a standard deviation of the Log R ratio above 0.35 were removed from the subsequent analysis (three samples). Furthermore, CNVs having less than five SNPs or a Log Bayes factor <10 were removed. The Log Bayes factor is a score that represents the support for the existence of the CNV and a Log Bayes factor of at least 10 will result in up to 10% false positive calls.

### Statistical and population genetics analysis

Prior to analysis, all chromosomes were centered on their mean log2 ratio to remove potential chip biases. The × chromosome was treated differently in this manner, since the pseudo-autosomal regions were centered separate to the rest of the chromosome, which differs in relative copy number to a male reference. To identify the pseudo-autosomal regions, the raw log2 ratios from all female samples were used to determine the average position of the probe where copy number changed from diploidy. Since no CNVs were successively called in that particular region, the position seems to be accurate.

The metric used to call CNVs from the aCGH data was the log2 ratio, which is the log (base 2) ratio of the observed normalized R-value for a signal intensity divided by the mean signal of a reference sample. The threshold of log2 ratio was defined as in the discovery and genotyping section to define a true copy number change that presents a pattern consistently different from a diploid region. CNVs were defined as |log2 ratio|>threshold, where deletions are indicated by negative log2 ratios and duplications are indicated by positive log2 ratios. Deletion and duplication variants are special cases of multi-allelic CNVs. To minimize the risk of false positives, we required each locus to be targeted by at least ten consecutive probes to call a CNV, resulting in a final resolution of approximately 9 kb.

Segmental duplications, defined as regions at least 1 kb in length, at least 90% identical at two or more loci, and not consisting entirely of mobile elements, were identified by self-alignment of the genome as described in Zody *et al*. [35]. In the GC peak analysis we identified GC peaks by sliding two windows along the genome; a 10 kb window for background rate (same size as a CNV breakpoint) and centered in this a 500 bp window for peak discovery. When the peak window showed a 1.5-fold increase in GC content compared to background, a GC peak was marked for all base pairs in the window. The program Neighbour from the Phylip package was used to construct the breed phylogeny from a distance matrix containing the pairwise differences between the breeds for all CNV loci.

GO analysis was made with the web-based tool g:GOSt Gene Group Functional Profiling provided by g:Profiler (formerly known as GOSt, Gene Ontology Statistics), [47], with default parameters. This tool not only searches for enrichment in GO categories, it also looks for enrichment in KEGG/REACTOME pathways and TRANSFAC regulatory motifs/MicroCosm microRNA sites as well as Human Phenotype Ontology and BioGRID protein-protein interaction networks. The set of orthologous genes was extracted with the tool g:Orth from the same project, which maps orthologous genes in related organisms using Ensembl alignments.

Statistical significance of overlap between genomic features was assessed by estimating the distribution of sample means. This was done by either random redistributions per chromosome or bootstrapping. Chromosome-wise redistributions were done by repeatedly and randomly redistributing all features on the same chromosome to get a distribution of means from which significance can be assessed. Bootstrapping was done by re-sampling the individual observations with replacement from the population of CNVs to get an empirical bootstrap distribution from which a bootstrap confidence interval could be derived to infer statistical significance.

R was used to smooth the aCGH data and the R package cghFLasso was used to assess copy number alterations. For genome annotation the University of Santa Cruz (UCSC) Genome Browser [48] and Ensembl Genome Browser [49] were used. Gene and exon coordinates were downloaded from Ensembl website version 65. Disease statuses of genes were obtained from the Online Mendelian Inheritance in Man database [50].

### Access to data

A database of genomic copy number variants, DoG_CNV, in the dog genome has been developed to provide the annotation of CNVs discovered in this study and a useful resource to assist with the assessment of CNVs in the contexts of canine variation and disease susceptibility [51]. In addition, the raw array data have been submitted to the Gene Expression Omnibus at NCBI [52].

## Abbreviations

aCGH: array comparative genomic hybridization; CNV: copy number variant; DSB: double-stranded break; GO: gene ontology; KEGG: Kyoto Encyclopedia of Genes and Genomes; LINE: long interspersed nuclear element; NAHR: non-allelic homologous recombination; qPCR: quantitative polymerase chain reaction; SD: segmental duplication; SINE: short interspersed nuclear element; SNP: single-nucleotide polymorphism.

## Competing interests

The authors declare that they have no competing interests.

## Authors' contributions

JB performed the majority of the bioinformatics analyses and drafted the manuscript. EMN performed the array experiments. A-MM analyzed the SNP array data. MP helped to coordinate the array experiments. CA participated in useful discussions. MCZ and TS identified segmental duplications. CH contributed bioinformatics analyses and constructed the website. KL-T and HL conceived of the study and participated in its design and coordination. MTW conceived and coordinated the bioinformatics analyses and wrote the paper. All authors read and approved the final version.

## Supplementary Material

Additional file 1 **Supplementary methods, tables and figures**. Click here for file
