# Supplementary material for: Novel origins of copy number variation in the dog genome
Source: Genome Biol. 2012 Aug 23;13(8):R73. doi: 10.1186/gb-2012-13-8-r73 (PMC4053742; doi:10.1186/gb-2012-13-8-r73)
Supplement: Additional file 1 — Supplementary methods, tables and figures. [file gb-2012-13-8-r73-S1.docx]

**SUPPLEMENTARY INFORMATION**

**SUPPLEMENTARY METHODS:**

We designed and optimised a method for genotyping CNVs consisting of three stages: 1) data smoothing, 2) segmentation algorithm and 3) CNV calling. The first stage, smoothing, is done to eliminate outliers and the random variation from the fluorescence trace data. The second stage, segmentation, is done by a new algorithm cghFLasso, which was chosen after comparison with four other methods (see below). Finally the third stage, calling, has also been improved. Instead of using a fixed threshold, it is now adjusted according to the standard deviation of the data (to better handle noisy samples).

**Step 1: Noise reduction (smoothing)**

To reduce noise in the data an 11-point triangular smoothing algorithm, centred on each probe, was applied before running the segmentation software. This method was aimed at compressing the data and especially the extreme outliers, while still maintaining the fine variation.

**Step 2: Segmentation algorithm**

In order to improve the calling of CNVs, we tested five segmentation algorithms. Data from the aCGH platforms (like NimbleGen) usually is log-ratios that represent the deviation in copy number from the normal state. Positive log-ratios indicate a gain and negative log-ratios indicate a loss in copy number.

A CNV calling algorithm works in two steps; first partitioning of the genome into suggested segments with differences in the deviation from normal, and second selecting segments that significantly differ from normal. One algorithm we evaluated (pennCNV) combines these two steps into one, only identifying segments with a significant deviation from normal, while the others leave the second step to the user, making manual tuning of the log-ratio threshold possible.

*Comparison of segmentation algorithms*

We evaluated five algorithms that each utilise different approaches for segmentation. This section gives a short outline for each of them. Where possible (all but pennCNV) a threshold of 0.45 was chosen. In the figures, altering shades of grey represent the segments, and the red ones represent the CNVs that are identified. The sample used as an example is from one of the Labrador Retrievers, which represents an average sample. We observed that the samples varied a lot in the amount of stochastic noise in the data.

*Method 1: NimbleGen*

Segments were identified using a Circular Binary Segmentation (CBS) algorithm implemented in the program segMNT, part of NimbleGen’s NimbleScan software, and the results can be seen in figure S1.

*Method 2: Ultrasome (http://www.broadinstitute.org/scientific-community/science/programs/cancer/ultrasome)*

This method that tries to minimize the probes’ squared error from the segment mean and also has a penalty constant to penalise the number of total segments. This can be set to optimize the method for detection of small or large aberrations. Results can be seen in figure S2. This method produces a large number of small segments that are unlikely to represent true CNVs.

*Method 3: DNAcopy (part of bioconductor R package http://www.bioconductor.org)*

Uses Circular Binary Segmentation (CBS) to detect regions with abnormal copy number. A recursive method that identifies change-points and tests for further change-points in the segments. See results in figure S3.

*Method 4: pennCNV (*www.openbioinformatics.org/penncnv/)

Implements a Hidden Markov Model that maximises the probability of being in a specific copy number state for each probe as well as the probability to change from that state based on the signal intensity that can integrate multiple sources of information to infer CNV calls. Results are found in figure S4. This method also leads to heavy segmentation.

*Method 5: cghFLasso (R package)*

Identifies DNA copy number alterations using the Fused Lasso method. It uses smoothing and derivatives to accurately capture both the piecewise flatness pattern and abrupt local jumps. See figure S5 for results.

Table S1 shows the average number of CNVs identified by the different methods in each breed. Some of the breeds consisted of samples that produced more noisy traces. For example, BTe, Box, Elk, FSp and GRe (marked in italics) contained such samples, and these breeds show more differences between methods. The cghFLasso algorithm performed best on the noisy data and showed a consistent number of CNVs independent of level of stochasticity in the traces. This method also produced the lowest standard deviation among samples. DNAcopy and Nimblegen also performed well in this respect, whereas pennCNV and especially ultrasome were very sensitive to noise in the samples.

**Step 3: Recalling the CNVs**

We chose cghFLasso, which produced most consistent results across samples, to perform segmentation. We ran it with the option of 0.05 FDR for segment identification. Once the segmentation is done comes the crucial step of picking thresholds for what is considered a copy number variant. This was performed in two stages: a) CNV locus identification and b) CNV calling (see methods in main text). We first identified CNV loci using a stringent threshold, followed by genotyping of CNVs at each loci using a less stringent threshold.

**Comparison of results**

We compared the results of our method with genotyping using the Nimblegen segmentation algorithm with arbitrary cut-off of 0.45 (Table S2). In the Nimblegen analysis, ~67% of the autosomal CNVs identified are specific to one breed with ~90% of those unique to a single sample. Using our method, ~20% of autosomal CNVs are breed specific and almost 50% of those shared within the breeds. Our method therefore identifies a much lower proportion of singleton CNVs.

We compared the number of CNVs identified per breed using our method with the number of SNP segregating per breed using the same samples run on the Illumina CanineHD SNP array. There was a highly significant correlation (Pearson's r=0.66; p<0.003). This is stronger than the next best method (Nimblegen r=0.60, p<0.008). The remaining methods (DNAcopy, pennCNV, and Ultrasome) did not produce significant correlations between levels of CNV and SNP diversity per breed. We also analysed the proportion of calls of each magnitude of deviation from the reference. A total of 25.8% of simple and 48.4% of complex CNV calls differed from reference, with the majority exhibiting calls consistent with single duplication or deletions (Figure S6).

**Table S1.** Average number of CNVs identified in each breed for the different methods. Italics marks particularly noisy breeds.

| breed | NimbleGen | cghFLasso | DNAcopy | pennCNV | Ultrasome |
| --- | --- | --- | --- | --- | --- |
| *BTe* | *104* | *106* | *290* | *640* | *1373* |
| *Box* | *61* | *53* | *233* | *790* | *1300* |
| CCS | 105 | 72 | 102 | 110 | 131 |
| Chi | 102 | 60 | 104 | 116 | 110 |
| Dac | 102 | 90 | 116 | 148 | 176 |
| ECS | 94 | 72 | 98 | 102 | 277 |
| ESS | 134 | 90 | 130 | 127 | 171 |
| *Elk* | *89* | *75* | *116* | *288* | *626* |
| *FSp* | *126* | *109* | *284* | *555* | *888* |
| *GRe* | *220* | *158* | *372* | *809* | *1785* |
| GSh | 66 | 57 | 65 | 75 | 145 |
| IrW | 180 | 107 | 148 | 146 | 326 |
| LRe | 89 | 58 | 106 | 222 | 181 |
| NSD | 107 | 70 | 109 | 121 | 172 |
| Pdl | 112 | 78 | 152 | 270 | 372 |
| Sar | 161 | 107 | 178 | 127 | 336 |
| Sch | 140 | 87 | 157 | 103 | 245 |
| Wlf | 78 | 57 | 98 | 221 | 235 |
| **Avg** | **115** | **84** | **159** | **276** | **492** |
| **S.D.** | **40.3** | **26.6** | **82.8** | **245.0** | **503.7** |

**Table S2.** Distribution of CNVs of different categories before and after applying the new method.

|  | multi-breed | | | breed-specific | | |  |
| --- | --- | --- | --- | --- | --- | --- | --- |
| breed | shared | unique | total | shared | unique | total | total CNVs |
| Nimblegen with fixed cut-off | | | | | | | |
| BTe | 36 | 43 | 79 | 2 | 23 | 25 | 104 |
| Box | 6 | 37 | 43 | 0 | 18 | 18 | 61 |
| CCS | 41 | 51 | 92 | 3 | 10 | 13 | 105 |
| Chi | 34 | 51 | 85 | 0 | 17 | 17 | 102 |
| Dac | 30 | 51 | 81 | 0 | 21 | 21 | 102 |
| ECS | 32 | 45 | 77 | 3 | 14 | 17 | 94 |
| ESS | 46 | 59 | 105 | 2 | 27 | 29 | 134 |
| FSp | 38 | 41 | 79 | 6 | 41 | 47 | 126 |
| GSh | 27 | 28 | 55 | 1 | 10 | 11 | 66 |
| GRe | 86 | 61 | 147 | 7 | 66 | 73 | 220 |
| IrW | 86 | 38 | 124 | 8 | 48 | 56 | 180 |
| LRe | 30 | 45 | 75 | 0 | 14 | 14 | 89 |
| NSD | 40 | 44 | 84 | 1 | 22 | 23 | 107 |
| Pdl | 20 | 68 | 88 | 2 | 22 | 24 | 112 |
| Sar | 40 | 56 | 96 | 3 | 62 | 65 | 161 |
| Sch | 32 | 60 | 92 | 3 | 45 | 48 | 140 |
| Elk | 24 | 41 | 65 | 1 | 23 | 24 | 89 |
| Wlf | 29 | 28 | 57 | 1 | 20 | 21 | 78 |
|  |  |  |  |  |  |  |  |
| new analysis | | | | | | | |
| BTe | 92 | 51 | 143 | 2 | 0 | 2 | 145 |
| Box | 24 | 69 | 93 | 0 | 1 | 1 | 94 |
| CCS | 97 | 74 | 171 | 3 | 3 | 6 | 177 |
| Chi | 98 | 70 | 168 | 0 | 3 | 3 | 171 |
| Dac | 77 | 80 | 157 | 0 | 8 | 8 | 165 |
| ECS | 84 | 66 | 150 | 0 | 1 | 1 | 151 |
| ESS | 100 | 89 | 189 | 1 | 5 | 6 | 195 |
| Elk | 64 | 79 | 143 | 1 | 0 | 1 | 144 |
| FSp | 96 | 66 | 162 | 5 | 3 | 8 | 170 |
| GRe | 186 | 49 | 235 | 5 | 2 | 7 | 242 |
| GSh | 82 | 64 | 146 | 2 | 1 | 3 | 149 |
| IrW | 177 | 21 | 198 | 11 | 1 | 12 | 210 |
| LRe | 84 | 69 | 153 | 0 | 2 | 2 | 155 |
| NSD | 91 | 81 | 172 | 1 | 2 | 3 | 175 |
| Pdl | 71 | 91 | 162 | 0 | 2 | 2 | 164 |
| Sar | 87 | 69 | 156 | 1 | 4 | 5 | 161 |
| Sch | 78 | 75 | 153 | 1 | 5 | 6 | 159 |
| Wlf | 82 | 67 | 149 | 0 | 2 | 2 | 151 |

CNVs are divided into those found in multiple breeds, or those that are breed-specific. These two categories can be further subdivided into categories whether they are shared among samples within the breed (shared) or found in only one sample in the breed (unique). The new analysis has reduced the number of CNVs that are unique to one sample, or to one breed.

**Table S3** Results of validation of CNVs on CanineHD SNP array

| CNVs | | | | | | |  |  |  |  |  |
| --- | --- | --- | --- | --- | --- | --- | --- | --- | --- | --- | --- |
|  | aCGH | | | HD | | |  |  |  |  |  |
| Chr | Start | Stop | Length | Start | Stop | Length | Array | non-ref | Ref | FP | FN |
| 4 | 33,735,718 | 33,839,190 | 103,472 | - | - | - | aCGH | 1 | 52 | 1 | 0 |
|  |  |  |  |  |  |  | HD | 0 | 53 |  |  |
| 5 | 89,133,635 | 89,408,025 | 274,390 | 89,137,039 | 89,424,540 | 287,501 | aCGH | 1 | 52 | 0 | 0 |
|  |  |  |  |  |  |  | HD | 1 | 52 |  |  |
| 7 | 16,215,120 | 16,351,851 | 136,731 | 16,250,630 | 16,331,860 | 81,230 | aCGH | 1 | 52 | 0 | 0 |
|  |  |  |  |  |  |  | HD | 1 | 52 |  |  |
| 12 | 56,324,020 | 56,677,655 | 353,635 | 56,309,275 | 56,673,236 | 363,961 | aCGH | 1 | 52 | 0 | 1 |
|  |  |  |  |  |  |  | HD | 2 | 51 |  |  |
| 13 | 59,905,884 | 60,330,228 | 424,344 | 59,911,694 | 60,321,923 | 410,229 | aCGH | 1 | 52 | 0 | 0 |
|  |  |  |  |  |  |  | HD | 1 | 52 |  |  |
| 17 | 44,660,727 | 44,788,924 | 128,197 | 44,662,229 | 44,820,120 | 157,891 | aCGH | 1 | 52 | 0 | 0 |
|  |  |  |  |  |  |  | HD | 1 | 52 |  |  |
| 20 | 19,148,116 | 19,405,125 | 257,009 | 19,155,699 | 19,399,607 | 243,908 | aCGH | 2 | 51 | 0 | 1 |
|  |  |  |  |  |  |  | HD | 3 | 50 |  |  |
| 21 | 10,531,634 | 10,964,721 | 433,087 | 10,531,606 | 10,959,977 | 428,371 | aCGH | 6 | 47 | 0 | 0 |
|  |  |  |  |  |  |  | HD | 6 | 47 |  |  |
| 21 | 40,562,554 | 40,743,475 | 180,921 | 40,580,407 | 40,742,936 | 162,529 | aCGH | 2 | 51 | 0 | 0 |
|  |  |  |  |  |  |  | HD | 2 | 51 |  |  |
| 26 | 34,270,582 | 34,691,538 | 420,956 | 34,274,763 | 34,684,997 | 410,234 | aCGH | 2 | 51 | 0 | 3 |
|  |  |  |  |  |  |  | HD | 5 | 48 |  |  |
| 28 | 42,193,308 | 42,405,924 | 212,616 | 42,191,398 | 42,437,874 | 246,477 | aCGH | 1 | 52 | 0 | 0 |
|  |  |  |  |  |  |  | HD | 1 | 52 |  |  |
| 32 | 13,130,573 | 13,401,082 | 270,509 | 13,154,284 | 13,378,328 | 224,044 | aCGH | 1 | 52 | 0 | 0 |
|  |  |  |  |  |  |  | HD | 1 | 52 |  |  |
| 33 | 5,628,605 | 5,886,383 | 257,778 | 5,642,316 | 5,887,595 | 245,279 | aCGH | 7 | 46 | 0 | 0 |
|  |  |  |  |  |  |  | HD | 7 | 46 |  |  |
| Total |  |  |  |  |  |  | aCGH | 27 | 662 | 1 | 5 |
|  |  |  |  |  |  |  | HD | 31 | 658 |  |  |

Comparison of the set of CNV loci identified from the CanineHD SNP array with calls from both aCGH and CanineHD. Coordinates from both the aCGH and CanineHD arrays are shown, along with the number of samples that match the reference or non-reference in both arrays. The FP column shows the number of calls that match reference in CanineHD but non-reference in aCGH (designated false positives). The FN column shows the number of calls that are non-reference in CanineHD but match reference in aCGH (designated false negatives). It should be noted that neither array is inherently more accurate, so lack of concordance does not necessarily indicate an error in the aCGH dataset.

**Table S4.** CNV breakpoint overlap with repeats.

| class | subfamily | average length (bp) | no. repeats in genome | no. repeats overlapping breakpoints | no. breakpoints overlapping repeats | obs/exp bases overlapping breakpoints |
| --- | --- | --- | --- | --- | --- | --- |
| Satellite | total | 677 | 1,524 | 31 | 10 | 8.38 |
| rRNA | total | 65 | 630 | 1 | 1 | 0.46 |
| RNA | total | 194 | 625 | 6 | 6 | 2.94 |
| LTR | ERV1 | 336 | 69,611 | 406 | 165 | 1.73 |
| LTR | ERVL | 304 | 90,102 | 393 | 216 | 1.34 |
| LTR | ERV | 317 | 460 | 2 | 2 | 1.24 |
| LTR | MaLR | 278 | 173,755 | 583 | 327 | 0.89 |
| LTR | total | 297 | 333,928 | 1,384 | 520 | 1.21 |
| LINE | L1 | 447 | 852,745 | 3,808 | 765 | 1.51 |
| LINE | CR1 | 186 | 45,699 | 112 | 90 | 0.76 |
| LINE | RTE | 198 | 13,800 | 34 | 27 | 0.61 |
| LINE | L2 | 226 | 323,386 | 881 | 426 | 0.71 |
| LINE | total | 377 | 1,235,630 | 4,835 | 832 | 1.36 |
| Simple repeat | total | 48 | 553,327 | 1,948 | 752 | 1.06 |
| snRNA | total | 62 | 4,645 | 16 | 16 | 1.06 |
| Low complexity | total | 41 | 392,820 | 1,365 | 616 | 1 |
| scRNA | total | 71 | 71 | - | - | - |
| SINE | Lys | 160 | 1,144,607 | 3,978 | 812 | 0.96 |
| SINE | MIR | 139 | 483,465 | 1,150 | 510 | 0.66 |
| SINE | total | 154 | 1,628,073 | 5,128 | 830 | 0.88 |
| tRNA | total | 61 | 2,038 | 7 | 7 | 1 |
| DNA | MER2_type | 319 | 33,223 | 90 | 56 | 0.68 |
| DNA | Tip101 | 219 | 22,754 | 63 | 42 | 0.9 |
| DNA | AcHobo | 180 | 15,952 | 56 | 45 | 0.86 |
| DNA | MER1_type | 174 | 172,278 | 491 | 285 | 0.83 |
| DNA | Tc2 | 204 | 6,400 | 13 | 9 | 0.71 |
| DNA | MER1_type? | 173 | 4,481 | 14 | 10 | 1.07 |
| DNA | MuDR | 60 | 134 | - | - | - |
| DNA | Mariner | 134 | 4,619 | 9 | 8 | 0.69 |
| DNA | DNA | 125 | 11,533 | 29 | 28 | 0.9 |
| DNA | piggyBac | 360 | 458 | - | - | - |
| DNA | total | 194 | 271,858 | 765 | 384 | 0.81 |
| Unknown | total | 185 | 894 | 1 | 1 | 0.29 |

**Table S5.** CNV breakpoint overlap with genomic features.

| Feature | Observed | Expected | Excess | p-value |
| --- | --- | --- | --- | --- |
| GC-peak | 269 | 124 | 2.2 | <0.001 |
| CpG-island | 263 | 173 | 1.5 | <0.001 |
| Gap | 205 | 147 | 1.4 | <0.001 |

Figures.

The figures S1-S5 below show variation in log2ratio for each probe along chromosomes from one sample, with one chromosome per row in increasing order from top to bottom and left to right. Segmentation is shown by alternating grey bars. Red bars represent segments with deviation >0.45 from the baseline, an arbitrary definition of a CNV.

**Figure S1.** NimbleGen segmentation for Labrador Retriever identifies 89 CNVs.


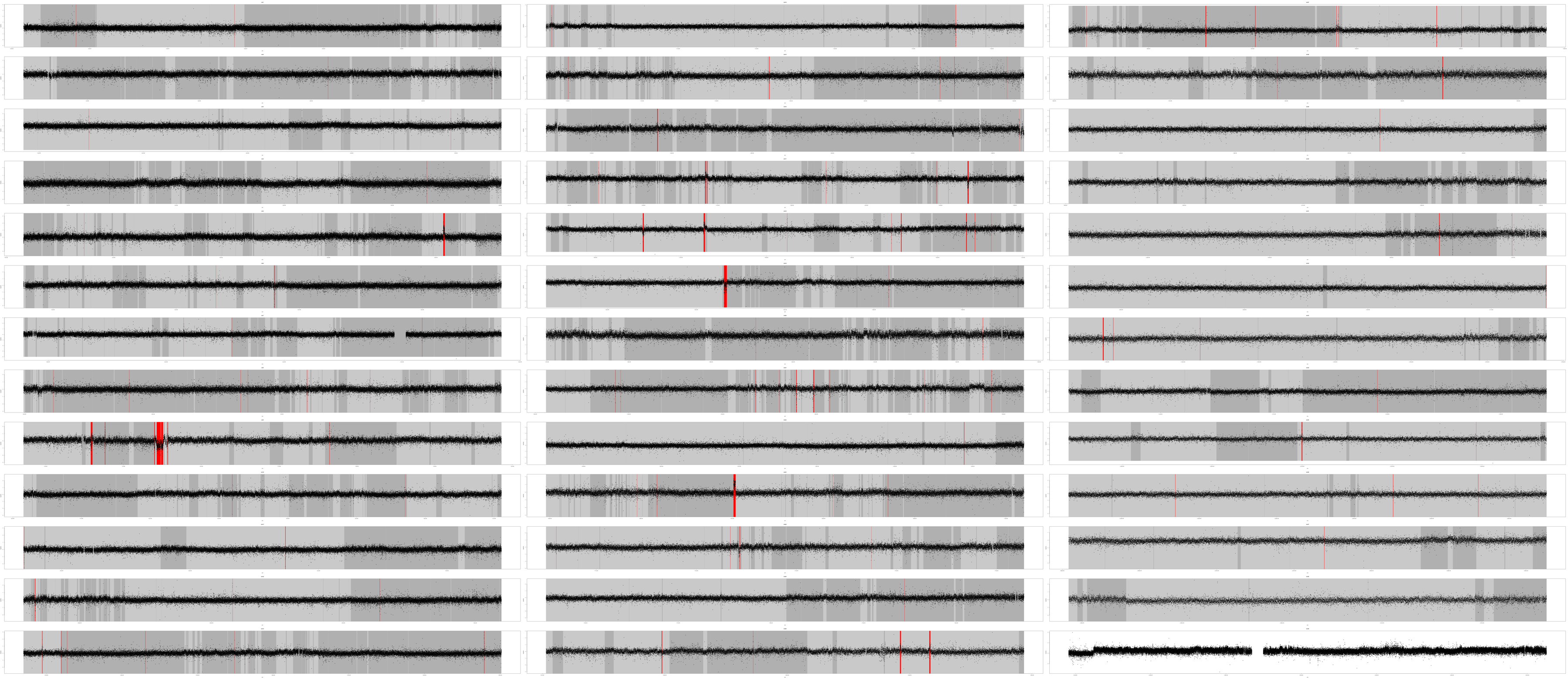


**Figure S2.** Ultrasome segmentation for Labrador Retriever identifies 181 CNVs.


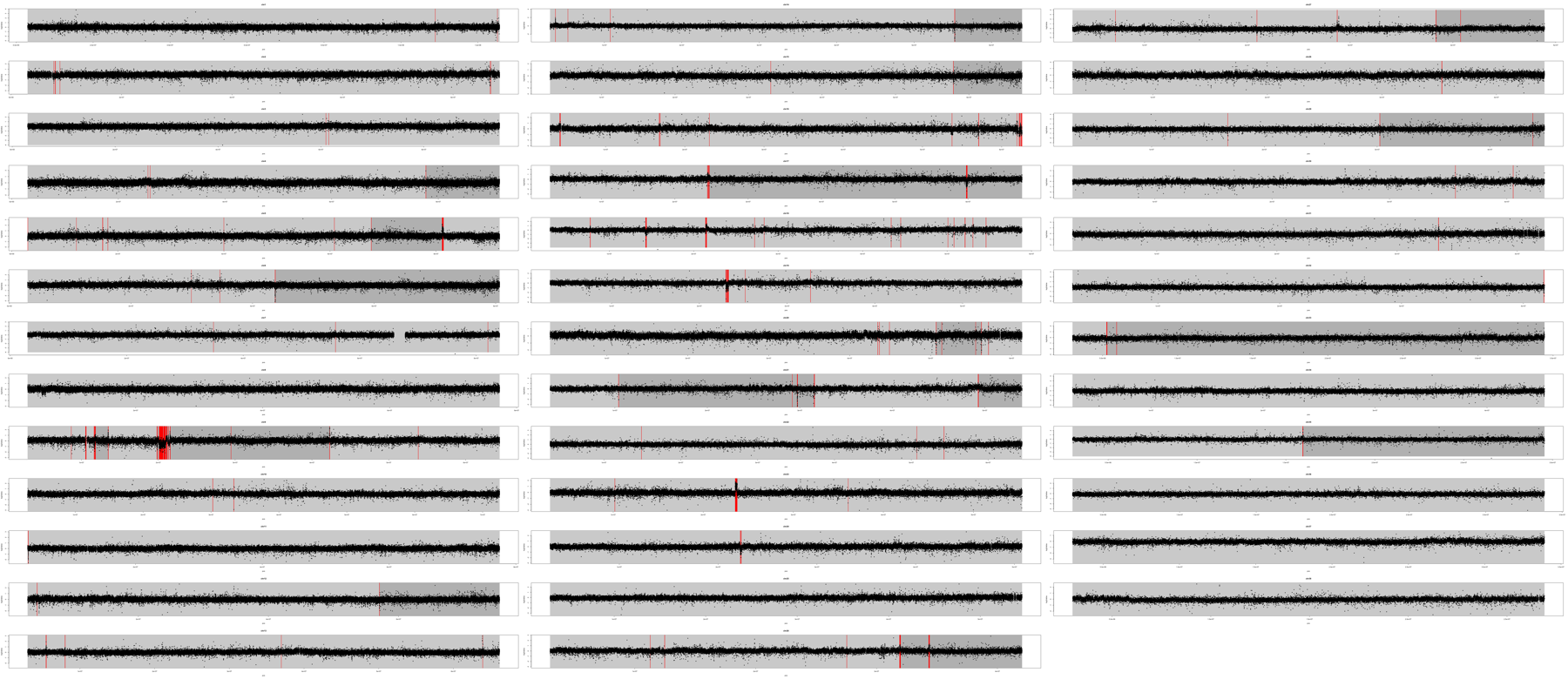


**Figure S3.** DNAcopy segmentation for Labrador Retriever identifies 106 CNVs.


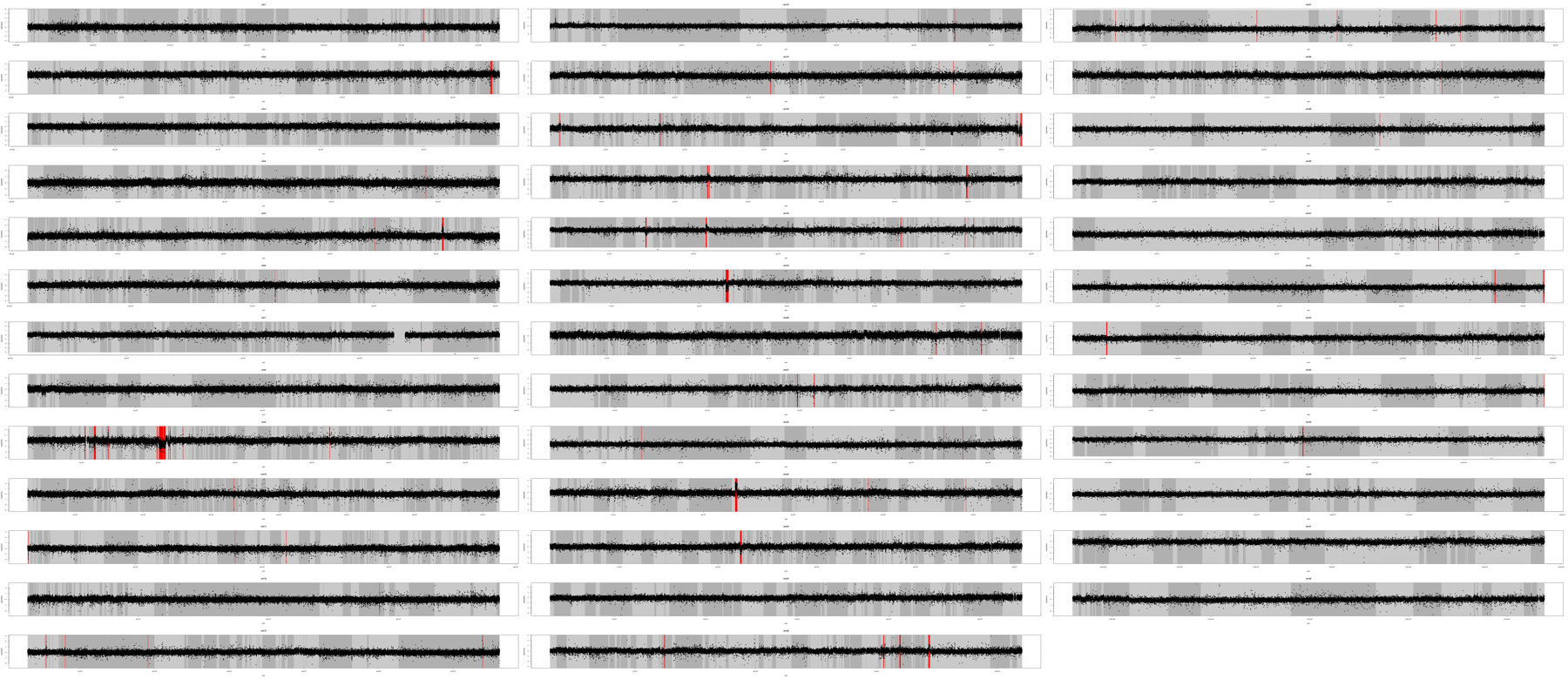


**Figure S4.** pennCNV segmentation for Labrador Retriever identifies 222 CNVs.


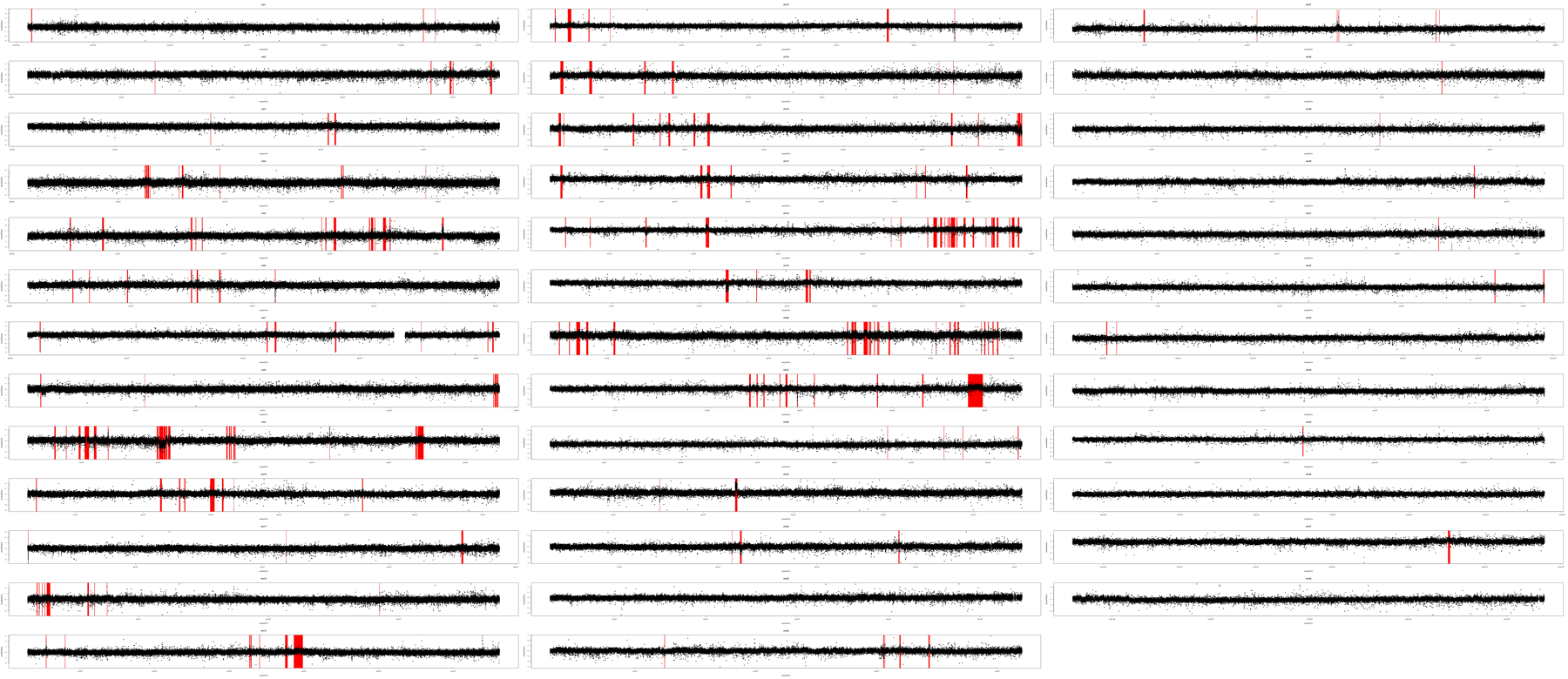


**Figure S5.** cghFLasso segmentation for Labrador Retriever identifies 58 CNVs.


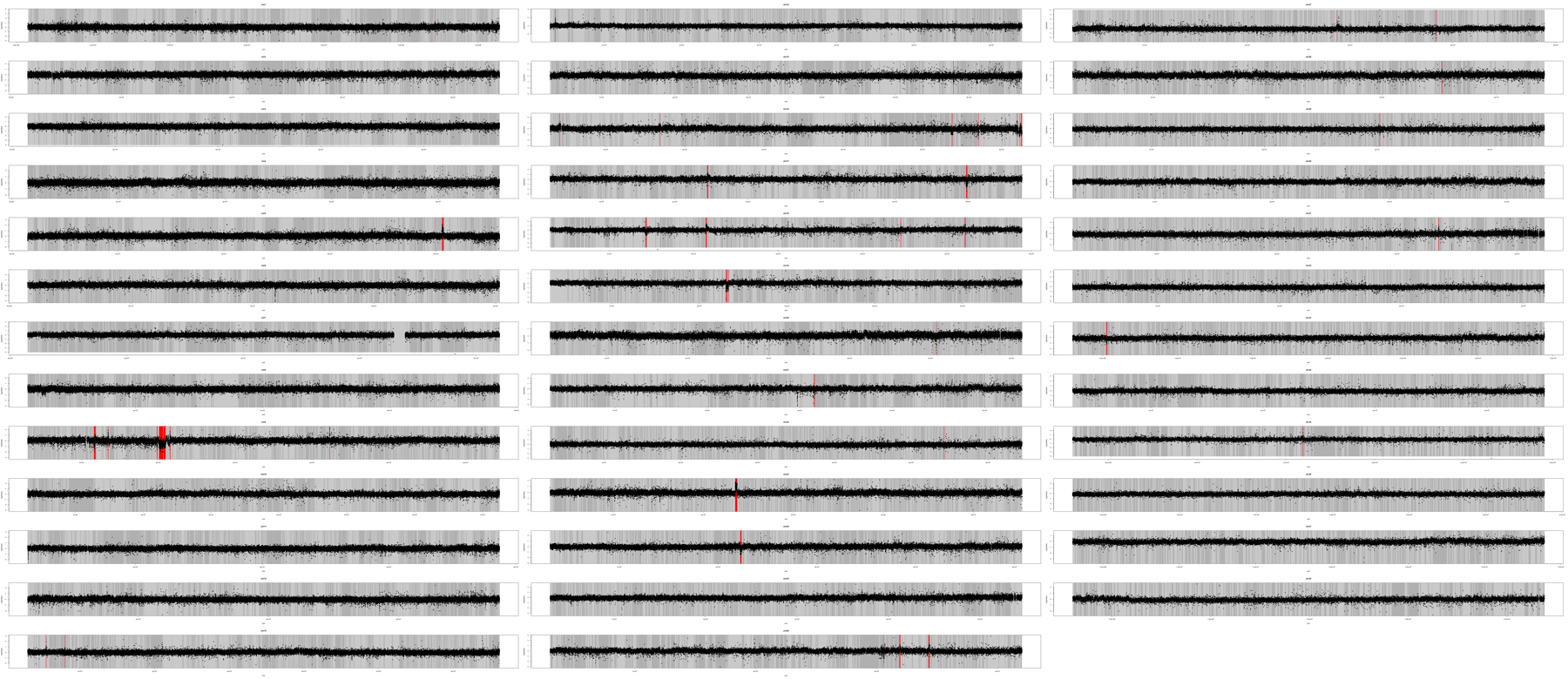


Figure S6. Total number of calls of each value in the dataset for simple and complex CNVs.
